# Editorial

**Published:** 1862-10

**Authors:** 


### Editorial.

N. Y. Ophthalmic School.—Dr. Mark Stephenson delivered the Introductory to his *Eleventh Course* of Lectures on Diseases of the Eye, at the New York Ophthalmic Hospital, cor. 4th Av. and 28th Street, on the 24th ult., to a large and attentive audience, composed of physicians and medical pupils from the several medical schools in the city.

He commenced by welcoming the students to the N. Y. Ophthalmic Hospital, and added he was happy to announce to them that the Institution, in whose behalf he appeared, was never in a more prosperous condition than at the present, averaging a thousand patients per annum, and numbering over three hundred graduates since its organization in 1852. 85 of whom were M.D.'s. After describing the requisite qualifications and duties of a surgeon, he cautioned them against attempting to make a display in their operations before a gaping crowd, for the purpose of exciting and dazzling an audience by the rapidity of their manipulations, as is too often apparent in some of our amphitheatres. He then drew an impressive contrast between the mere operator and the scientific and skilful surgeon.

Spoke next of the eye, anatomically and pathologically—of its organization and delicacy of structure, transcending all that is wonderful in design and admirable in execution.

That as the eye or its appendages were composed of all the different tissues which enter into the composition of the human frame, so its diseases were more numerous than that of any other organ in the body; also the effect of treatment, whether operative or otherwise, was more satisfactory than that of any other class of diseases, from the fact that there are so many objective symptoms; contrasted the treatment of former times, when Thompson's eye water was the sovereign remedy, with the enlightened and scientific methods of the present day, proving clearly that no department of medical literature had made greater strides in modern days than ophthalmic medicine and surgery. He endeavored to impress upon the minds of his hearers the importance of attending to this department of the profession; especially those intending to practice in the country, where adequate counsel is difficult to be obtained. Hence the importance of their availing themselves of the untold advantages to be derived from attending the cliniques and lectures of an ophthalmic school; that this branch of the profession may no longer be suffered to fall into the hands of empirics and charlatans, to the disgrace of regular practitioners. The time had now come when the stigma should be wiped from the profession, and no longer rest as a reproach upon surgeons, in the middle of the nineteenth century. Every medical man, claiming to be an accomplished physician and surgeon, should be well acquainted with ophthalmic medicine and surgery; urged them not to despond in view of the vastness of the undertaking; what others had accomplished and overcome they might.

He finally closed by saying: "Gentlemen, think nobly of your profession. Remember that its end is *beneficent*, its studies *ennobling and elevating*, its ministration an exercise of your best faculties. To excel in it is worthy of all your aspirations and energies; but requiring mental and moral discipline, patient and persevering labor. Make of your difficulties a school in which strength of character may be tried. There is no sweeter reminiscence than that of difficulties overcome. Cultivate a love of knowledge, for the benefit it will enable you to confer upon others, then what ever worldly fortune betide, you will win the most valuable of all blessings which the occupation of a life can confer, accomplish the will of your Maker and live to His glory."

---

Illinois State Medical Society.—Shall we have a meeting of the State Medical Society at the regular time next May? Two regular annual meetings have been postponed, on account of the continuance of civil war, and the large number of members absent with our volunteer army; and we raise the question in relation to a further postponement thus early, to elicit from those interested, a free expression of opinion.

It cannot be denied, that repeated postponements are apt to terminate in the permanent dissolution of all voluntary social organizations. Hence all who desire a continuance of our State organization should favor the holding of the next meeting at the appropriate time. Jacksonville, the place selected for the next meeting, is an attractive locality. Several interesting and important State institutions are located there, and will be accessible to the members of the Society. We trust the meeting will not only be held, but that it will be largely attended also, by delegates from all parts of the State.

---

CHICAGO MEDICAL SOCIETY.—The regular meetings of this Society are held on Friday evening of each week, in Larmon's Block, on the corner of Clark and Washington Streets. They are well attended, and the exercises interesting and profitable.

---

APPOINTMENT.—The Governor has filled a vacancy in the Board of Medical Examiners for this State, by the appointment of DANIEL BRAINARD, M.D., Professor of Surgery in Rush Medical College.

---

### CHICAGO MEDICAL SOCIETY.—Nov. 7, 1862.

Regular monthly Meeting. Dr. WICKERSHAM in the chair. Minutes of last meeting read and approved.

Dr. TUCKER exhibited a large pedunculated tumor, fibro-polypoid in character, which he had removed from the posterior and left side of the cavity of the neck of the uterus. The tumor had existed 18 years. A few days since, the patient was attacked with severe expulsive pains, when the tumor was suddenly forced from the uterus, and remained suspended from the vulva. Dr. TUCKER was called for the purpose, as the patient stated, of having her womb replaced. An examimation revealed the true nature of the tumor, which was cut away, after the pedicle had been ligated.

Dr. TUCKER also related the case of a woman æt. 32, to whom he was called in her third confiement. He found a child delivered and dead; the placenta lying in the vagina and mouth of the uterus. Upon placing one hand on the abdomen, he could distinctly feel a round, hard body like the uterus partially contracted as after delivery; he could also feel another child lying above the umbilicus across the abdomen. On passing the hand into the vagina, to remove the placenta, two distinct and separate mouths of the uterus were discovered; one large, from which the placenta was removed, and the other smaller and closed. The woman recovered in the usual length of time, the ordinary discharge of lochia occurring. More than two months have elapsed since the first birth took place; the second and remaining child continues to develop, and will probably be delivered in about six weeks.

Dr. Holmes moved that Dr. Tucker be requested to visit the patient from time to time, and to report the result at some future time.

Dr. Holmes reported the case of a cyst, which he had removed from the breast of a young woman, 17 years of age. The only point of particular interest in the case consisted in the peculiar form of the cyst, which was about an inch long, and a-third of an inch in diameter, and penetrated deep into the gland.

Dr. Hatch opened the discussion on Scarlatina anginosa, followed by Drs. Holmes, Tucker, Waite, Davis, Paoli, and Peterson.

It was voted that the meetings of the Society be held every Friday evening, through the winter and spring.

Question for discussion at next meeting—Camp Diarrhœa.

Adjourned. E. L. HOLMES, *Sec'y.*

---

Affections of the Throat in Scotland.—Sore-throat, ulcerated sore-throat, and diphtheria, have occurred in various localities in Scotland, and in Mid and South Yell. The sore-throat appears to have been accompanied with an affection of the hands, which raises the suspicion that sore-throat and diphtheria in the human subject is but a variety of the epidemic disease in cattle known by the name of murrain or epizootic aptha, characterized by the aphthous and ulcerated mouth and sore hoofs.

